# Spatial heterogeneity of tree diversity response to climate warming in montane forests

**DOI:** 10.1002/ece3.7106

**Published:** 2020-12-28

**Authors:** Ting Li, Peng Luo, Qinli Xiong, Hao Yang, Xiaodong Gu, Yuming Qiu, Bo Lin, Yang Liu, Changhong Lai

**Affiliations:** ^1^ Key Laboratory of the Evaluation and Monitoring of Southwest Land Resources (Ministry of Education) Sichuan Normal University Chengdu China; ^2^ CAS Key Laboratory of Mountain Ecological Restoration and Bioresource Utilization & Ecological Restoration Biodiversity Conservation Key Laboratory of Sichuan Province Chengdu Institute of Biology Chinese Academy of Sciences Chengdu China; ^3^ State Key Laboratory of Urban and Regional Ecology Research Center for Eco‐Environmental Sciences Chinese Academy of Sciences Beijing China; ^4^ The Wildlife Protection Division of the Forestry Department of Sichuan Province Chengdu China; ^5^ Chengdu Institute of Computer Applications Chinese Academy of Sciences Chengdu China; ^6^ Chongqing Institute of Green and Intelligent Technology Chinese Academy of Sciences Chongqing China; ^7^ Sichuan Forestry and Grassland Research and Planning Institute Chengdu China

**Keywords:** climatic warming, montane forests, species nestedness, species turnover, tree diversity

## Abstract

Many studies reported biotic change along a continental warming gradient. However, the temporal and spatial change of tree diversity and their sensitivity to climate warming might differ from region to region. Understanding of the variation among studies with regard to the magnitude of such biotic changes is minimal, especially in montane ecosystems. Our aim is to better understand changes in spatial heterogeneity and temporal dynamics of mountain tree communities under climate warming over the past four decades. In 2017, we resurveyed and recorded all tree species from 107 long‐term monitoring plots that were first studied between 1974 and 1976. These plots were located in montane forests in the Giant Panda National Park (GPNP), China. Our results showed that spatial differences were found in tree species diversity changes response to mean annual temperature change over the past four decades. Tree species richness increased significantly under climate warming in Minshan (MS) and Xiaoxiangling (XXL) with higher warming rate than Qionglai (QLS) and Liangshan (LS). The trees species diversity in MS and XXL were more sensitive to climatic warming. MS and XXL should receive priority protection in the next conservation plan of the GPNP. The GPNP should avoid taking a “one‐size‐fits‐all” approach for diversity conservation due to spatial heterogeneity in plant community dynamics.

## Graphical abstract

Tree species richness and abundance of montane forests increased over time in all our study area, except Liangshan (LS), especially in XiaoXiangLing with the highest warming rate. However, beta diversity underwent a significantly higher change rate at LS than in other mountains which indicated that plant species that do not belong to these four mountains entered LS in those years. Moreover, the beta diversities of tree between sample plots in the LS regions were homogenized.

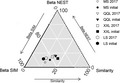

## 1 INTRODUCTION

Climate change affects ecosystems both directly and indirectly in a number of nonuniform ways (Milad et al., 2011). Since most organisms, at both fine and coarse spatial scales, differ significantly in their response to climate change, large spatial scale models cannot reliably determine the impact of altered climates on vegetation (Milad et al., 2011; Xiong, Halmy, et al., 2019; Xiong, Xiao, et al., 2019). This is particularly true since species also differ in their abundances, functional traits, distributions, and habitat associations at various scales (Baltzinger et al., 2011; Li, Xiong, Luo, Zhang, et al., 2020). Nevertheless, this spatial variation in plant diversity responses to climate change still remains relatively unknown, particularly for montane forest ecosystems (Li, Xiong, Luo, Zhang, et al., 2020).

The forest ecosystem is dominated by long‐lived perennials plants (Dakhil et al., 2019; Xiong, Halmy, et al., 2019). Previous studies have shown inconsistent results for these perennials plants. For example, losses of cold‐adapted tree species under warming on boreal‐temperate mountains in Europe appear to have been very few (Kulonen, 2017). On the other hand, climate warming can also drive increases of regional perennials plant species richness (Xiong et al., 2016). We only have very few understanding of the processes of shifting in plant diversity that underlie this variation because of lacking of long‐term monitoring sample fileds and slowly responding of plants, espically tree speices. However, temperature at high elevations in many mountain ranges increased faster than the World average (Alexander et al., 2018). Such rapid changes in temperature resulted in a dramatic turnover in alpine plant communities (Hülber et al., 2016; Xiong et al., 2016). Since most previous studies monitored plant communities in short temporal scales, resurveying the plots might provide insight on the temporal changes that montane forests endure on larger temporal scales (Becker‐Scarpitta et al., 2019; Kapfer et al., 2017; Nielsen et al., 2019). Historical biodiversity data can provide a baseline against which to measure changes. However, most of these studies focused on a single site or region that occurred within the time frame in which the data were collected (Newbold et al., 2015; Xiong, Xiao, et al., 2019). Therefore, it is urgent to increase the knowledge of changes of biodiversity in large spatial scales in long term (Kulonen, 2017; Xiong et al., 2016).

Projections using ecological niche models (ENMs) predict up to 100% species turnover in alpine plant communities in some region by climate warming in late 21st century (Dakhil et al., 2019; Engler et al., 2011). Climate is capable of large effects on plant species turnover (Zellweger et al., 2017). Beta diversity is a measure of the change in species composition across over time or space (Tisseuil et al., 2012); it is a better indicator than alpha diversity for the trends of changes in plant community structure and composition under climate change in multiscale (Svenning et al., 2011). For this, in‐depth studies of beta diversity are helpful to understand biomes and to protect plant biodiversity under climate warming. Still, much biodiversity conservation research uses alpha diversity as the key assessment criterion, leaving beta diversity far less explored, especially in mountain areas (Vasconcelos et al., 2018).

The Giant Panda National Park (GPNP) with a total area of 27,000 km^2^ (over 80% of the area is mountain region) in China was established in 2017. The ambition of the GPNP to preserve the Giant Panda habitat is self‐evident (Li, Xiong, Luo, Zhang, et al., 2020). However, the GPNP management plan does not provide clear measures to mitigate climate change (Sichuan Provincial Government, 2017). While considering the complex and highly heterogeneous mountainous landscape that characterizes the GPNP, management strategies likely need to be region‐specific, since the impact of climate warming on such a large conserved area might differ from region to region. The question of whether and how to incorporate spatial heterogeneity into the management of GPNP is therefore a pressing one.

Here, based on repeated surveys of forest tree communities in montane forests in the GPNP in areas covering a range of recent climate warming trends (Zhang, Mathewson, Zhang, Porter & Ran, 2018) and local meteorological data, general linear mixed‐effect models (GLMMs) were used to analyze changes of trees species alpha and beta diversities in response to climate warming over the past 40 years. The aim was to address two key questions: What changes have occurred in trees species alpha and beta diversities in response to climate warming over the past four decades? How did these changes differ on a regional scale? Here, we hypothesized that (a) climate warming may lead to a slight increase in tree species richness, and species shift upwards higher elevations, while beta diversity may decline in our study area; (b) species richness would increase notably in some regions with higher warming rate, where the rate of diversity change may be different from other regions. In the process of protecting biodiversity, this study will provide a reference for the spatially different management of mountain ecosystems under climate warming.

## 2 MATERIALS AND METHODS

### 2.1 Study regions

The study regions comprised four mountainous sites in Sichuan Province, China: Minshan (MS), Qionglai (QLS), Xiaoxiangling (XXL), and Liangshan (LS) mountains, all of which are located in the transitional region between the Qinghai–Tibet plateau and the Sichuan basin. The climate of the study area is a typical mountain climate based on the subtropical monsoon climate, shows significant vertical difference, and the climate change among regions is complex and sensitive (Li, Xiong, Luo, Zhang, et al., 2020; Sichuan Vegetation Cooperation Group, 1980). The mean annual temperature is 10.0–15.0°C, the mean temperature in January (coldest month) is −6.0–1.0°C, and the mean temperature in July (warmest month) is 11.0–17.5°C. The four mountains are located in the rainy zone of West China (Sichuan Vegetation Cooperation Group, 1980). The annual precipitation in the study area is about 550–1,250 mm (Table 1).

**Table 1:** the climatic characteristics of Minshan (MS), Qionglai (QLS), Xiaoxiangling (XXL), and Liangshan (LS) mountains in study area (Sichuan Vegetation Cooperation Group, 1980)

Mountains	Mean annual temperature (°C)	Annual precipitation (mm)	The number of plots	The range of elevation (m)
MS	5.7–13.5	550–820	32	2,150–3,192
QLS	13.0–15.0	≥1,200	43	2,100–3,192
XXL	10.7–16.6	800–1,250	11	2,080–3,496
LS	10.0–11.6	1,000–1,200	21	2,200–3,636

The most prevalent ecosystems are cold and warm temperate coniferous forests or broad‐leaved mixed forests with more than 800 wild vertebrates and 4,000 wild vascular plants in those four mountains (Dakhil et al., 2019; Li, Xiong, Luo, Zhang, et al., 2020; Sichuan Vegetation Cooperation Group, 1980). In terms of trees species abundance, the plants in the study area mainly consist of *Abies* sp., *Picea* sp., *Betula* sp., *Tsuga* sp., and *Sabina* sp.

### 2.2 Study plots

**Figure 1:**
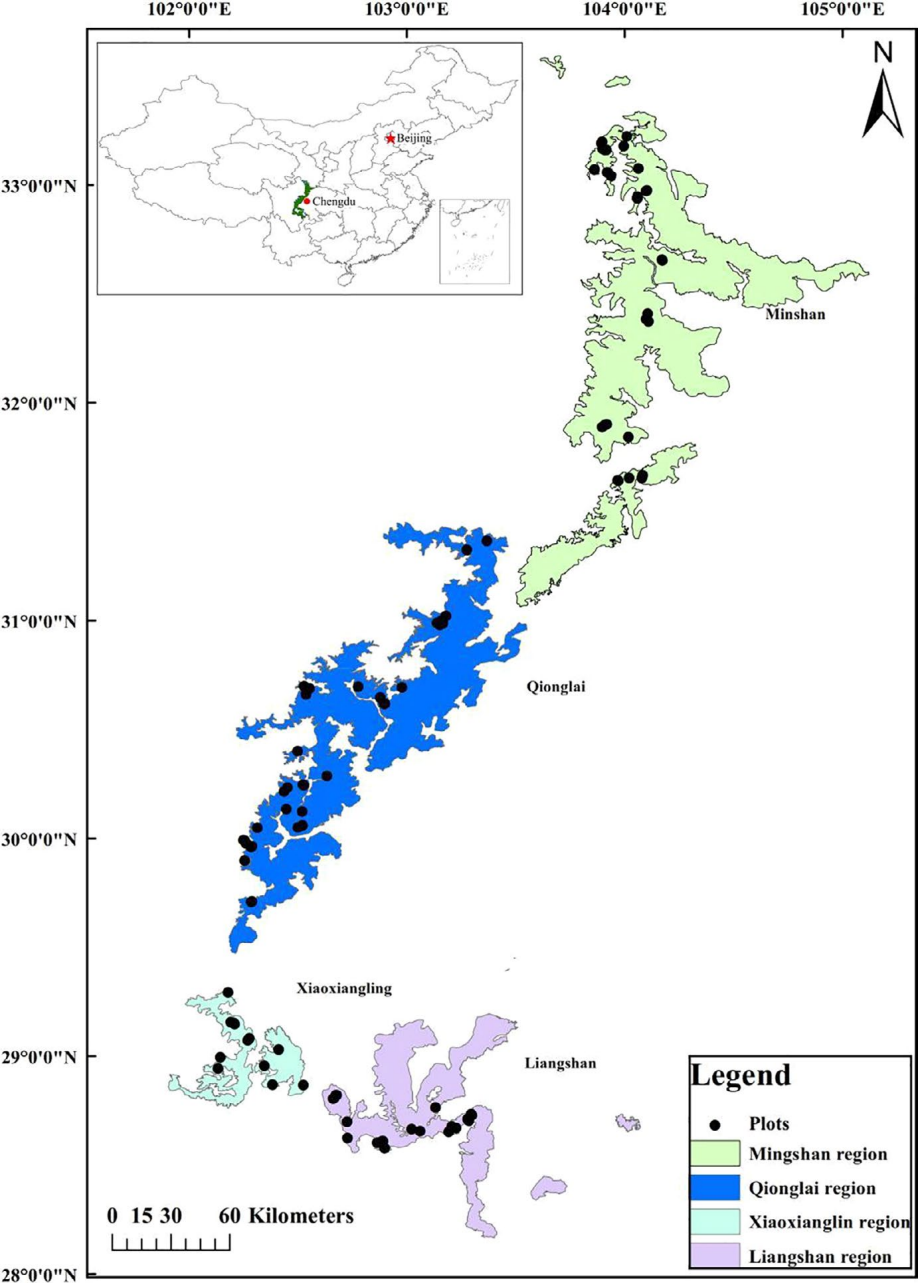
Distribution of the revisited plots of the Giant Panda National Park (GPNP, Sichuan Forestry Department provided boundaries)

The study plots were first surveyed by the Chengdu Biology Institute of the Chinese Academy of Sciences in 1974–1976, which were since revisited and resurveyed using the same methods in 2017 (Table S1). The initial plots were marked during the first survey. Several historical plots have not been found for historical reasons, and several plots experienced human or natural disturbances (e.g., felling, insect outbreaks, fire, or earthquake) and were excluded in our study. In several historical plots, we screened repeated plots following criteria: (a) virgin forests or mature natural forest plots in GPNP; (b) excluded historical plots where land‐use patterns had changed, and where two earthquakes in 2008 and 2013 had effects on study sites; (c) excluded plots with human interference; and (d) random sampling. A total of 107 plots (20 m × 30 m) were selected among many long‐term monitoring locations (Figure 1). These 107 plots were mostly located in temperate coniferous or broad‐leaved mixed forests located on slopes of high mountain areas at elevations between 2,000 and 3,600 m (Table S1). The elevation data of each plot were obtained by field measurement.

Within each plot (20 m × 30 m), we resurveyed and recorded all tree species, shrub, and herb in 2017. Trees with diameter at breast height ≥ 10 cm at height 1.3 m were used for this study.Vegetation surveys were conducted between July and September in each survey year (the peak period of plant growth). We invited plant taxonomy professionals to identify plant species. In the field, 99% of plants were identified to the genus level and 90% to the species level. For plants that were not known in the field, we collected and produced plant specimens and photos to the herbarium of Chengdu Institute of Biology, Chinese Academy of Sciences, for identification. Plant taxonomy and nomenclature were thoroughly standardized, using broad species concepts to avoid false appearances/disappearances.

### 2.3 Meteorological data

We obtained meteorological data from in situ meteorology stations in each study region between 1974 and 2017. We collected monthly and annual temperature data in 14 weather stations in past four decades. Meteorological data are provided by the Sichuan Meteorological Bureau, the Forestry Bureau, and the Environmental Protection Agency and Water Affairs Bureau. In addition, several research institutes have meteorological monitoring stations in natural reserves, such as Jiuzhaigou and Baodinggou Nature Reserves, where CIB, CAS has established a long‐term monitoring station in the 1980s. For each weather station, monthly meteorological data were interpolated to a spatial resolution of 30 m × 30 m for the whole study area, and climatic driver factors of each plot were estimated by using the Kriging interpolation method which was widely used for regionalizing various variables at different scales (Piao et al., 2011; Xiong et al., 2020; Xiong, Xiao, et al., 2019; Zhu et al., 2006). According to the Sichuan Statistical Yearbook and Meteorological Bureau historical records in the study area during past 40 years, no pest outbreaks, or fires, had occurred in sample plots during the 40‐year study period from 1974 to 2017.

### 2.4 Data analysis

#### 2.4.1 Tree beta diversity in the four mountains

We classified 107 plots based on their location in the four mountains (MS, QLS, XXL, and LS), and used the Sorensen index (presence–absence) to characterize the pairwise dissimilarity of species composition in the four mountains. The Sorensen index was divided into turnover (beta SIM) and nestedness (beta NEST) components to investigate whether the tree species beta‐diversity component would change under climate change (Baselga, 2010). As used here, turnover represented the replacement of tree species, while nestedness represented the gain/loss in tree species (Baselga, 2010). We also calculated the Sorensen‐based similarity index (1‐Sorensen). To present the variation in species composition over time and space, this triplet of response variables (beta SIM, beta NEST, and similarity) was plotted in a triangular graph for the four mountains from 1974 to 2017.

#### 2.4.2 Spatial differences in tree species diversity change rates among different mountains

To fairly compare compositional/diversity changes among the four mountains, we first calculated the annual change rate of tree richness, tree abundance, and beta diversity indicator (=the net change rate over time within local areas) as (*x*_resurvey_ − *x*_initial_)/*t*, where *t* represents the census interval (in years) and *x* represents the value of the response variable of interest at resurvey and initial survey. We check for normality of each variable. An ANOVA (LSD test, least significance difference) was performed using the RSTAT2D package v1.0 in R v3.4.3 to multiple compare significant differences of each variable change among mountains.

#### 2.4.3 The sensitivity of tree species diversity responses to mean annual temperature change in different mountains

Factors that affect plant diversity change included mean annual temperature (MAT) change, elevation and other factors such as nitrogen deposition, precipitation, and microbe diversity in our study area (Xiong et al., 2016). However, previous study found that climate warming is one of the most important drivers of vegetation change in this area (Zang et al., 2017). We used mean annual temperature (MAT) to analyze the relationship between tree species diversity (richness, abundance, beta, beta SIM, and beta NEST) and climate warming.

To gauge the sensitivity of plant communities to MAT change, and to identify the most sensitive regions, we used a general linear mixed‐effect model (GLMM), with a Poisson error structure, to quantify the relationship between species richness and abundance per mountain. We included “MAT × elevation” as a fixed explanatory variable, and “plot” in each mountain as a random variable.

(1) $$Y_{i}=a_{0}+\;a_{1}\times {\text{MAT}}_{i}+a_{2}\times {\text{Elevation}}_{i}+a_{3}\times {\text{MAT}}_{i}:{\text{Elevation}}_{i}+{\text{plot}}_{i}+e_{i}$$

where *i* = 1, 2, …, 64 in MS, 1, 2…, 86 in QLS, 1, 2…, 22 in XXL, and 1, 2, …, 42 in LS: *a_0_* is your random intercept term, *a_1,_ a_2,_* and *a_3_* represent the regression coefficients, and *e_i_* represents the residual vector. The response variable, *Y_i_*, represents either tree richness or abundance in *plot_i_* during the initial year or 2017.

We analyzed the relationship between trees species beta diversity (beta, beta SIM, beta NEST) and MAT change difference in different plots from the initial year to 2017. We used a GLMM, with a binomial error structure, with the following form:

(2) $$Y_{\mathrm{ij}}=a_{0}+a_{1}\times \;\text{Distance}‐{\text{MAT}}_{\mathrm{ij}}+a_{2}\times \;\text{Distance}‐{\text{elevation}}_{\mathrm{ij}}+a_{3}\times \text{Distance}‐{\text{MAT}}_{\mathrm{ij}}:\text{Distance}‐{\text{elevation}}_{\mathrm{ij}}+{\text{site}}_{i}+{\text{site}}_{j}+e_{\mathrm{ij}}$$

where *i* ∈ 1, 2, …, 31; *j* ∈ 2, 3, …, 32 in MS; *i* ∈ 1, 2, …, 42; *j* ∈ 2, 3, …, 43 in QLS; *i* ∈ 1, 2, …, 10; *j* ∈ 2, 3, …, 11 in XXL; and *i* ∈ 1, 2, …, 20; *j* ∈ 2, 3, …, 21 in LS. The response variable, *Y_ij_*, is beta, beta SIM, or beta NEST, with “*Distance‐MAT_ij_ *× *Distance‐elevation_ij”_* as fixed explanatory variate. *Distance‐elevation_ij_* = |H_*i*_ − H_*j*_|, H, elevation; *Distance‐MAT_ij_* = |MAT_*i*_ − MAT_*j*_| (Li & Waller, 2017). We added plot*_i_*, plot*_j_* as random factors. Meanwhile, we analyzed the variation of alpha and beta diversity with MAT change in overall GPNP from the initial year to 2017. All models were validated following suggestions by Zuur, Ieno, Walker, Saveliev & Smith, (2009). The GLMM analysis was performed in the glmmTMB package (v3.1‐137) in R v3.4.3.

## 3 RESULTS

### 3.1 Differences in change rate of plant diversity (richness, abundance, beta diversity, beta SIM, and beta NEST) among mountains

**Figure 2:**
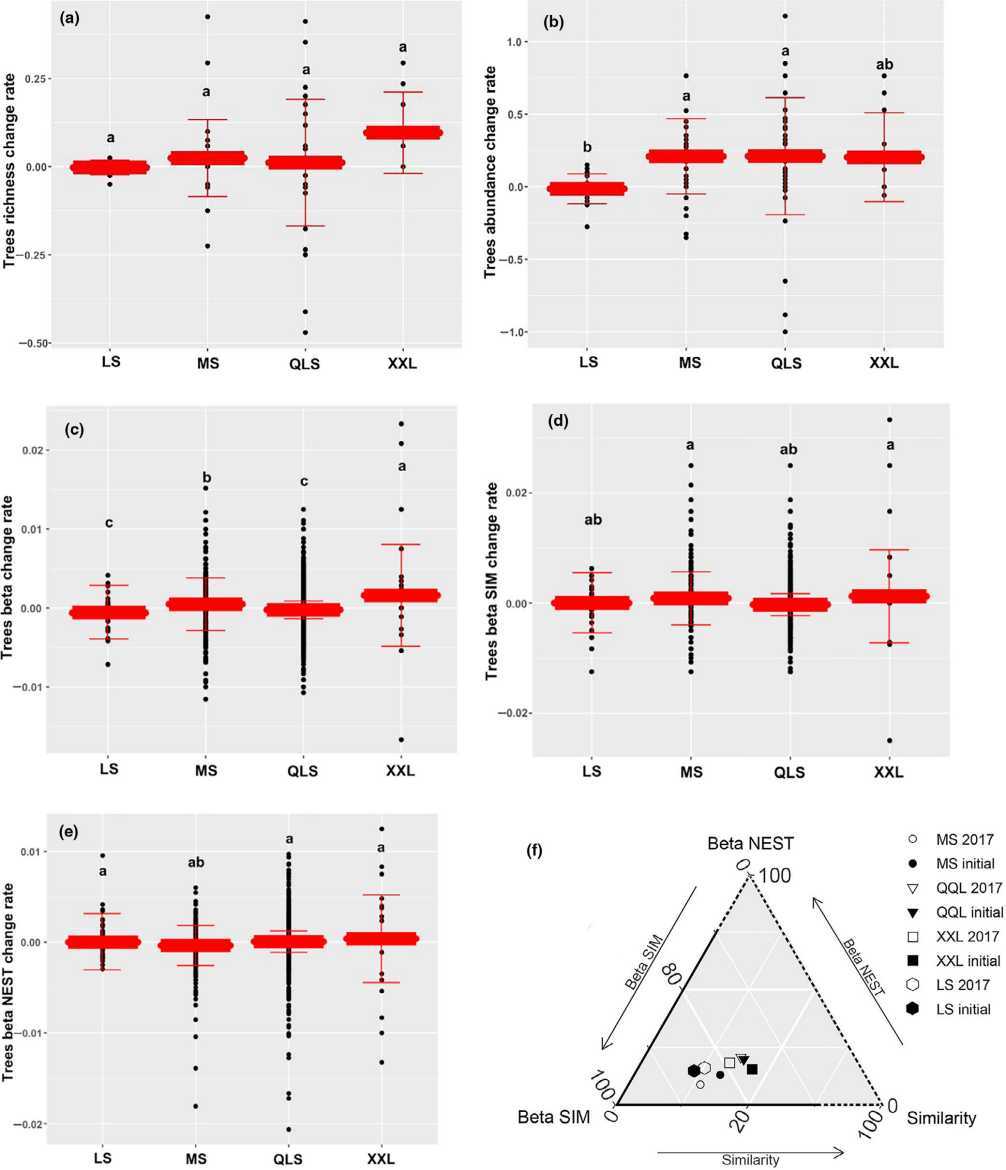
Results of ANOVAs on change rates of (a) tree richness, (b) abundance, (c) beta, (d) beta SIM, and (e) beta NEST compared among the four mountains. The solid middle line represents the median, and the box represents the interquartile range of the coefficients. Whiskers extending to extreme data points and outliers are points that exceed 1.5 times of the interquartile range from the interquartile range. (f) Ternary plots showing the values of different dissimilarity in different mountains in the initial year and 2017. The Sorensen dissimilarity coefficients were decomposed to turnover (beta SIM) and nestedness (beta NEST) components. The dots are centroids of the respective mean values of the beta SIM, beta NEST, and similarity components. MS, Minshan; QLS, Qionglaishan; XXL, Xiaoxiangling; LS, Liangshan

Tree species richness increased from the initial survey year to 2017 in all mountains (except for LS, which showed a slight decrease (Figure 2a)); however, the differences among mountains were not significant. Tree abundance increased significantly at MS, QLS, and XXL in the past four decades, while it decreased slightly in LS (Figure 2b). The change rate of abundance was significantly higher at MS, QLS, and XXL than at LS (*F*_3,103_ = 3.88, *p* = .039), which exceeded the change rate of species richness over time.

However, beta diversity underwent a significantly higher change rate at XXL than in other mountains (Figure 2c) (*F*_3,103_ = 4.60, *p* = .0033), and was significantly lower in QLS and LS versus the other two mountains. The beta SIM changing rates of XXL and MS were significantly higher than those of QLS and LS (Figure 2d) (*F*_3,103_ = 3.31, *p* = .020), thus indicating higher tree species turnover at XXL and MS. In contrast, the beta NEST change rate was similar among the four regions (Figure 2e). Beta SIM dominated the Sorensen index (Figure 2f).

### 3.2 The effects of mean annual temperature change on tree species diversity in different mountains

**Figure 3:**
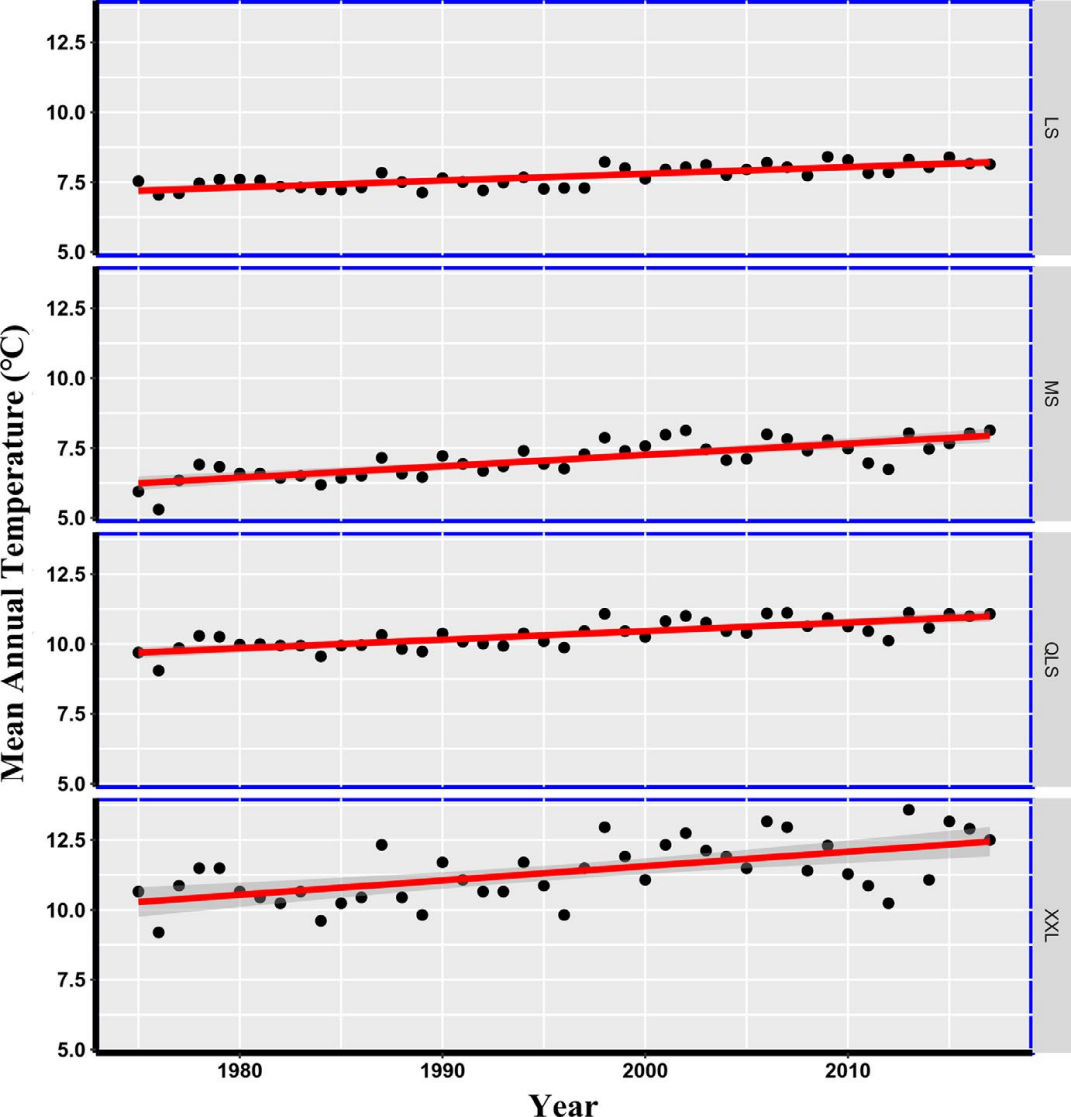
The change of mean annual temperature (°C) in different mountains over time. MS, Minshan; QLS, Qionglaishan; XXL, Xiaoxiangling; LS, Liangshan

**Figure 4:**
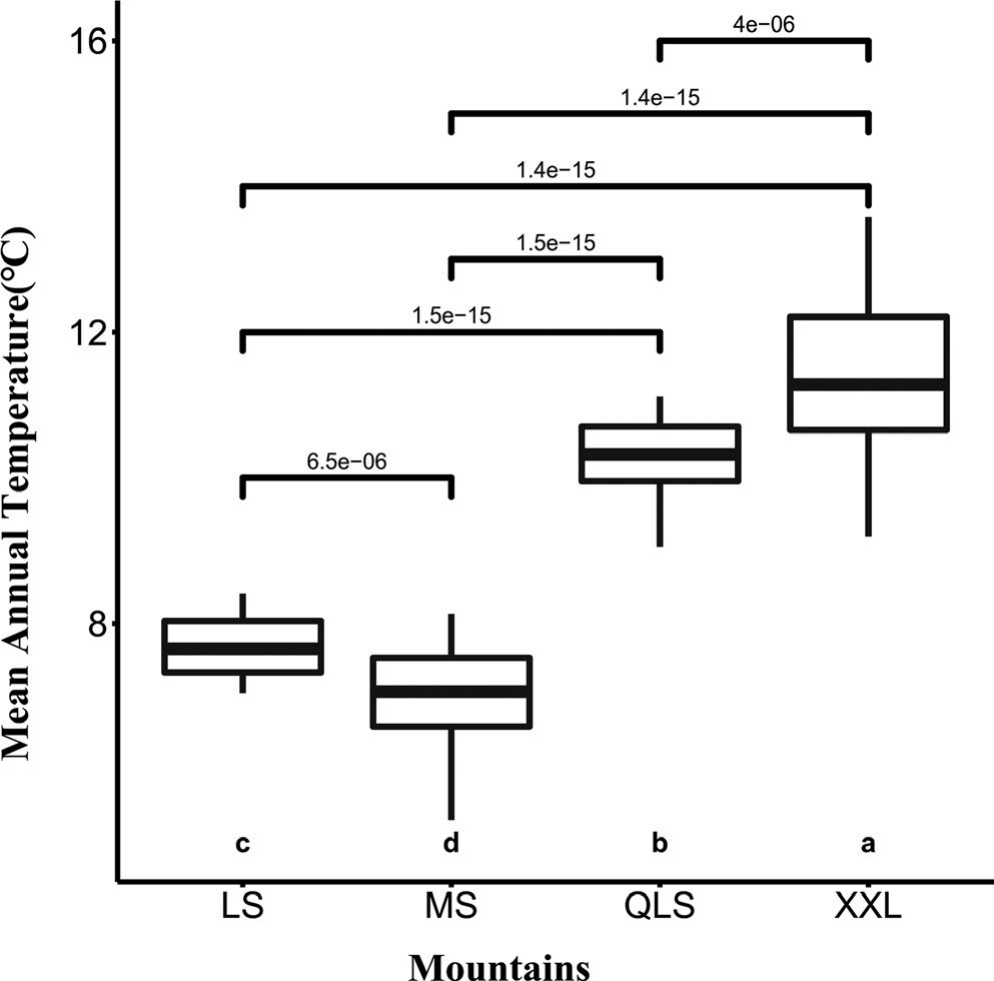
Results of ANOVAs mean annual temperature (°C) in different mountains. MS, Minshan; QLS, Qionglaishan; XXL, Xiaoxiangling; LS, Liangshan. The a, b, c, and d represent the level of significant difference

Figure 3 shows that the MAT in the study area has increased significantly since the initial survey, at an annual warming rate of 0.034°C/year. Nonetheless, annual warming rates differed among the four mountains, and were fastest at XXL (0.051°C/year), followed by MS (0.040°C/year), then QLS (0.031°C/year), and slowest at LS (0.024°C/year). The mean annual temperature of the four mountains shows a significant difference. The highest absolute value of MAT is in XXL, and the lowest one is in MS (Figure 4).

Table 2 shows the spatial differences in plant species diversity in response MAT change in the four mountains. In overall GPNP, warming had significant positive effect on species richness (*Z* value = 2.48, *p* = .013); interaction effect of MAT change and altitude had significant (*Z* value=−2.47, *p* = .013) negative effect on richness; and beta nest significantly decreased (*Z* value = −2.01, *p* = .044) with *Distance* ‐ *MAT* variation in the past four decades. In MS, species richness increased significantly with MAT change (*Z* value = 3.16, *p* = .0016) and altitude (*Z* value = 2.69, *p* = .0071); interaction effect of MAT and altitude had significant (*Z* value = −3.14, *p* = .0017) negative effect on richness. In XXL, species richness increased significantly with MAT change (*Z* value = 2.15, *p* = .032) and altitude (*Z* value = 2.04, *p* = .042); interaction effect of MAT and altitude had significant (*Z* value = −2.03, *p* = .042) negative effect on richness. These results showed that tree species richness of MS and XXL were more sensitive to MAT change and altitude than other two mountains. In overall GPNP, richness and beta NEST were sensitive to MAT change.

**Table 2:** Summary of tree species alpha and beta diversity (absolute values) rnesponses to climate warming, based on a generally linear mixed model (GLMM)

Mountains	Index	Fixed effects					Random effects
		MAT	H	MAT:H	Intercept	*df*	
All habitat	Richness	21.88*	3.2	−6.37*	−10.18	208	Plots
	Abundance	4.13	0.60	−1.20	−0.58	208	
	Beta diversity	0.0015	−0.0023	0.0014	0.28***	8,474	Pair plots
	Beta SIM	0.017	−0.00030	−0.0015	0.26***	8,474	
	Beta NEST	−0.020*	−0.0027	0.0043	0.033***	8,474	
MS	Richness	57.49**	12.89**	−16.66**	−43.72**	58	Plots
	Abundance	4.71	0.57	−1.36	−0.47	58	
	Beta diversity	0.015	0.0036	−0.012	0.27***	714	Pair plots
	Beta SIM	0.033	0.0067	−0.022	0.25***	714	
	Beta NEST	−0.024	−0.0037	0.013	0.023	714	
QLS	Richness	−6.28	−6.09	1.75	22.03	80	Plots
	Abundance	11.28	2.04	−3.26	−5.64	80	
	Beta diversity	0.040	0.0084	−0.019	0.25***	1,338	Pair plots
	Beta SIM	0.041	0.0084	−0.019	0.25***	1,338	
	Beta NEST	−0.052	−0.0091	0.022	0.053***	1,338	
XXL	Richness	381.76*	116.66*	−104.18*	−426.71*	16	Plots
	Abundance	380.91	121.75	−107.38	−430.24	16	
	Beta diversity	−0.79	−0.0034	0.24	0.29	74	Pair plots
	Beta SIM	−3.48	0.035	0.98	0.24	74	
	Beta NEST	3.08	−0.043	−0.84	0.056	74	
LS	Richness	34.00	7.07	−9.95	−23.33	36	Plots
	Abundance	−4.16	−0.66	1.04	4.21	36	
	Beta diversity	−0.046	−0.0050	0.013	0.29***	304	Pair plots
	Beta SIM	−0.12	−0.0085	0.041	3.11***	304	
	Beta NEST	0.12	0.0057	−0.043	0.0084	304	

Note

H represents elevation for richness and abundance, which represents *Distance‐elevation* for beta diversity.

MAT represents mean annual temperature for richness and abundance, which represents *Distance‐MAT* for beta diversity.

MS, Minshan; QLS, Qionglaishan; XXL, Xiaoxiangling; LS, Liangshan; *df*, degrees of freedom.

For the GLMM, *indicate the significance of fixed effects (***p < .001, **p < .01, *p < .05).

## 4 DISCUSSION

The analysis of this study focused on the impacts of mean annual temperature change on spatial plant diversity over 40 years. The aim was to better understand spatial and temporal dynamics of mountain tree community change under climate warming in a long term. Robust analyses that acknowledge the complexity and heterogeneity of outcomes at different scales and locations should provide the strongest case for a strategic outlook. The results were mostly consistent with our predictions, and the magnitude of richness changes most often increased in XXL and MS, where the warming trends have been stronger. Trees species richness increased along elevations in XXL and MS.

### 4.1 Climate warming improved tree diversity increasing

Collectively, the mean temporal change of tree species diversity increased in the past four decades, whereas ecosystems undergoing postdisturbance succession will often show increases in richness over time (Vellend et al., 2013; Xiong, Halmy, et al., 2019). Environmental factors such as climatic warming and deterministic processes such as environmental filtering should exert a greater effect on biological diversity than stochastic processes (Guo et al., 2018; Victorero et al., 2018). Recently, Berteaux et al. (2018) predicted that although climate change can drive increases of regional species richness. Moreover, since warmer areas tend to have higher local plant diversity than cold areas, climate warming has been predicted to increase local plant diversity (Vellend et al., 2017). To a certain extent, our study result confirms this prediction richness indeed increased in MS and XXL with greater warming rate (Figure 3, Table 2). This indicates that trees species richness of these montane forests increased in response to climate warming when the MAT has risen to a certain point. It should be noted that the effect of extreme temperatures in plant mountain communities did not consider in our study. Further research to identify those effects for better knowing the relationship between climate change and shift of biodiversity is needed.

### 4.2 trees species may migrate upward along elevation

As predicted, some mountain trees species richness increased under warming in MS and XXL with higher warming rate than LS and QLS. Then, the interaction effect of MAT and altitude had significant negative effect on richness, which suggest some maladaptive species may migrate upword along elevation, even disappear in the upper limit of forest distribution under climatic warming. The increased richness under warming may come from two sources: lower elevation species shifted up to the elevation, replenished the lost species, and adapting warming new species replaced and replenished the lost species in greater numbers than the lost species. Because the climatic warming has a negative effect on beta NEST, some original maladaptive species might migrate out of the forest distribution line, have been lost. The range of altitudes (2,000–3,700 m) we study plots is fixed, and it may include the upper limit of forest distribution, but not the lowest limit of forest distribution.

As the study show, climate warming has resulted in a significant upward shift in species optimum elevation averaging 29 m per decade in west Europe (Lenoir et al., 2008). Because trees species are at least partially spatially tracking their temperature optima in response to warming (Sproull et al., 2015). Of course, there are differences in the upward migration of trees species among the four mountains, which may be due to more than just different warming rates, for example, topographic, plant species composition, soil organic matter content, and microbial properties (Mayor et al., 2017). Warming would direct and indirect affect trees migrating along elevations (Li, Xiong, Luo, Zhang, et al., 2020), which could disrupt the functional properties of montane ecosystems (Svenning & Sandel, 2013) and result in periods of disequilibrium where range shifts may be compensated for by species from lower latitudes and faster population turnover (Lenoir et al., 2008).

Turnover refers to a species replacing other species, independent of changes in local species richness. In nature, species turnover may reflect species sorting by the environment or in response to dispersal dynamics (Qian, 2010; Qian et al., 2010). Nestedness suggests that reductions or increases in species richness are nonrandom and ordered extinction–colonization dynamics (Siqueira‐Souza et al., 2016), and both the number and variation of turnover and nestedness are closely related to the local environment. We found that climatic warming significantly affected the plant community composition (beta NEST) in entire GPNP, yet its richness increased significantly, suggesting that tree species in lower elevtion shifted upward, or other new species were recruited to offset losses of original species. Here, no significant change was found in established nor dominant species, implying the loss of rare species only. The emerging patterns of species extinctions with increasing drought and temperature likely resemble the pattern of extinctions in order of species abundance, where rare species are the first to be extirpated (Memmott et al., 2004). This was supported by Gray et al. (2016), who suggested that protected areas are effective for species richness but did not play a prominent role for the protection of rare and endemic species. There are rare/endemic populations of trees in GPNP, including *Tetracentron sinense* sp., *Davidia involucrate*, *Taxus chinensis*, and *Magnolia sinensis*, among others. Southwestern China is an important refuge for *Tetracentron sinense* sp. and *Davidia involucrata*, which were likely shaped by both pre‐Quaternary and Pleistocene climatic changes (Sun et al., 2015; Xiong, Xiao, et al., 2019). However, the main adaptive management goals of current conservation policies are to expand the coverage of the protected area and establish regular patrols coupled to decentralized household monitoring (Cao et al., 2017). None of these methods can help to protect rare species (Brockerhoff et al., 2013) in GPNP. It should be noted that those rare species that played decisive roles in the process of beta‐diversity change were not identified in our study, and therefore, further research to identify their involvement is needed.

### 4.3 New conservation policy: Management measures for warming sensitive regions

In our study, plant diversity and community composition response to climate warming differed at different regions in space; XXL and MS are climate sensitive regions (Table 2, Figure 3). This is consistent with work by Dornelas et al. (2014), who concluded that a spatial difference exists in the direct impact of climate change on plant richness on a global scale. Contemporary conservation policies do not cover the complete region or all species occurring in this mountain ecosystem (Table 1, Figure 2), thus likely leaving rare plant populations and regions (e.g., XXL and MS) still at risk. It is still necessary to adopt a new sustainable conservation policy for the GPNP that specially targets its at‐sensitive regions. It is worth remembering that there is no “one‐size‐fits‐all” approach (Brockerhoff et al., 2013).

To protect the diversity and structural integrity of the XXL and MS plant communities, the following actions should be implemented: (a) carry out distribution and population surveys of rare plants, and identify species that are particularly vulnerable to climate warming, so that appropriate conservation plans can be developed; (b) monitor the effectiveness of various protected areas under the influence of climate change, and develop better optimized and targeted protection management measures instead of simply expanding the protected area; and (c) expand the focus of research and planning from biodiversity loss toward a change of biodiversity and species' compositions.

Admittedly, this study did not consider economic factors of the new conservation policy; hence, it ignores the potential costs and benefits of such a policy for the GPNP. In reality, conservation activities are directed by either the government or by local individuals. Due to explicit budgetary constraints, these decision makers will most likely consider the cost and potential economic value of any conservation project.

## 5 CONCLUSIONS

Spatial differences were found in montane forest tree species beta‐ and alpha‐diversity changes in response to mean annual temperature change over the past four decades. Stronger warming led to more changes in species richness in our study, especially them in XXL and MS, which may become sensitive regions under continuing climatic warming, and should thus receive priority protection in the next conservation plan of the GPNP. The GPNP should avoid taking a “one‐size‐fits‐all” approach for diversity conservation due to spatial heterogeneity in plant community dynamics. We must also emphasize accounting for plants' compositional changes when focusing on their diversity loss. This study improved our understanding of climate change effects on spatial and temporal dynamics of subalpine biodiversity in montane forests. Equipped with this knowledge, conservation priorities that jointly maintain biodiversity and habitat integrity can better be identified.

## CONFLICT OF INTEREST

None declared.

## AUTHOR CONTRIBUTIONS

**Ting Li:** Conceptualization (equal); data curation (equal); formal analysis (equal); investigation (equal); methodology (equal); writing–original draft (equal); writing–review and editing (equal). **Peng Luo:** Funding acquisition; writing–original draft; writing–review and editing (equal). **Qinli Xiong:** Conceptualization (equal); funding acquisition (equal); writing–original draft (equal); writing–review and editing (equal). **Hao Yang:** Software (equal); writing–original draft (equal); writing–review and editing (equal). **Xiaodong Gu:** Writing–review and editing (equal). **Yuming Qiu:** Writing–review and editing (equal). **Bo Lin:** Writing–review and editing (equal). **Yang Liu:** Resources (equal). **Changhong Lai:** Resources (equal).

## Supporting information

Table S1

## Data Availability

Data from this study are archived in the public archive Dryad Data (https://datadryad.org/) at the Repository. https://doi.org/10.5061/dryad.w6m905qmj (Li, Xiong, Luo, Gu, et al., 2020).
