# Supplementary material for: Spatial heterogeneity of tree diversity response to climate warming in montane forests
Source: Ecol Evol. 2020 Dec 28;11(2):931–41. doi: 10.1002/ece3.7106 (PMC7820142; doi:10.1002/ece3.7106)
Supplement: Supplementary file 1 — Table S1 [file ECE3-11-931-s001.DOCX]

Table S1 Plots descriptions

| Moun  tains | Site code | Protected area(ha) | Plot  numbers | plot  area(ha) | Coordinates | Plots’ altitude (m) | Dominant species | MAP (mm) | | MAT (℃) | | No. giant panda | | No. Plant Species | |
| --- | --- | --- | --- | --- | --- | --- | --- | --- | --- | --- | --- | --- | --- | --- | --- |
|  |  |  |  |  |  |  |  | 1975 | 2017 | 1975 | 2017 | 1985 | 2015 | Initial | 2017 |
| Min  shan | Jiuzaigou | 64297 | 11 | 0.66 | 103.8-104.0E  33.0-33.2N | 2292-3192 | *Abies, Picea wilsonii* | 714 | 1088.70 | 5.17 | 7.40 | 7 | 3 | 95 | 83 |
|  | Wanglang | 32297 | 12 | 0.72 | 104.0-104.1E  32.3-32.9N | 2150-3050 | *Abies, Betula, Picea* | 849.9 | 741.70 | 4.70 | 6.70 | 19 | 28 | 97 | 92 |
|  | Baodinggou | 89884 | 9 | 0.54 | 103.8-104.0E  31.6-31.9N | 2160-3060 | *Abies, Tsuga,* [*Betula*](http://frps.eflora.cn/frps/Betula) | 919.5 | 702.20 | 7.96 | 10.30 | / | 35 | 109 | 105 |
| Qiong  lai | Wolong | 200000 | 14 | 0.84 | 103.1-103.3E  30.9-31.3N | 2300-3027 | *Abies, Betula* | 1021.25 | 895.95 | 7.73 | 9.80 | 72 | 104 | 105 | 112 |
|  | Jiajingshan | 39039 | 10 | 0.6 | 102.4-102.9E  30.4-30.6N | 2350-3192 | *Cercidiphyllum, Acer* | 2037.40 | 1641.10 | 8.59 | 9.10 | 23 | 38 | 96 | 91 |
|  | Labahei | 23437 | 10 | 0.6 | 102.3-102.6E  30.0-30.2N | 2100-3000 | *Abies, Tsuga,* | 1911.34 | 1532.36 | 10.40 | 9.00 | 2 | 20 | 96 | 96 |
|  | Erlangshan | 57648 | 9 | 0.54 | 102.2E  29.7-29.9N | 2400-3140 | *Abies, Quercus* | 745.00 | 664.00 | 12.07 | 13.40 | / | 19 | 83 | 71 |
| Xiaoxiangling | Lizhiping | 47940 | 11 | 0.66 | 102.1-102.4E  28.8-29.1N | 2080-3496 | *Abies, Tsuga,* [*Betula*](http://frps.eflora.cn/frps/Betula)*, Sabina* | 1451.37 | 1250.00 | 10.65 | 12.50 | / | 22 | 63 | 71 |
| Liang  shan | Dafengding | 50655 | 10 | 0.6 | 103.02-103.3E 28.57-28.86N | 2200-3000 | *Abies, Picea, Castanopsis* | 1135.0 | 840.0 | 7.51 | 8.47 | 23 | 22 | 91 | 93 |
|  | Shengguozhuang | 33700 | 11 | 0.66 | 102.5-102.8E  28.5-28.8N | 2360-3636 | *Abies, Betula* | 1033.20 | 1021.80 | 7.56 | 7.80 | / | 2 | 62 | 65 |
|  | Total | 638897 | 107 | 6.32 | 102.1-104.1E  28.5-33.2N | 2080-3636 |  |  |  |  |  | 146 | 293 |  |  |
